# Maternal HFD and CDAHFD Exposure from Pregnancy to Lactation Differentially Affect Lipid Profiles and Behavior in Offspring Mice

**DOI:** 10.3390/nu18213589

**Published:** 2026-10-29

**Authors:** Ryoken Aoki, Nobuo Izumo, Megumi Furukawa, Rena Ikeda, Chisato Takiya, Ayaka Ito, Hideo Matsuzaki

**Affiliations:** 1 Department of Functional Brain Activities, United Graduate School of Child Development, University of Osaka, Suita 565-0871, Osaka, Japan; r.aoki@yok.hamayaku.ac.jp; 2 Center for Pharmaceutical Education, Yokohama University of Pharmacy, 601 Matano-cho, Totsuka-ku, Yokohama 245-0066, Kanagawa, Japan; 3 Research Center for Child Mental Development, University of Fukui, 23-3, Matsuokashimoaizuki, Eiheiji 910-1193, Fukui, Japan; 4 Laboratory of Pharmacotherapy, Yokohama University of Pharmacy, 601 Matano-cho, Totsuka-ku, Yokohama 245-0066, Kanagawa, Japan; megumi.furukawa@yok.hamayaku.ac.jp (M.F.); yhtrh15@gmail.com (R.I.); chiisney1342@gmail.com (C.T.); conan.aya91041@gmail.com (A.I.); 5 General Health Medical Research Center, Yokohama University of Pharmacy, 601 Matano-cho, Totsuka-ku, Yokohama 245-0066, Kanagawa, Japan

**Keywords:** offspring, triglyceride, open field, dopamine

## Abstract

Background/Objectives: Maternal nutritional status during pregnancy and lactation can influence metabolic and neurobehavioral development in offspring. However, the contribution of specific lipid alterations to these effects remains unclear. This study investigated whether maternal exposure to different high-fat diets produces distinct metabolic and neurobehavioral outcomes in offspring. Methods: Pregnant ICR mice were fed a control diet, a conventional high-fat diet (HFD), or a 50% choline-deficient, amino acid-defined high-fat diet (50% CDAHFD) from embryonic day 12 until weaning. Male offspring were assessed at 4–6 weeks of age for body weight, plasma lipid levels, behavioral phenotypes, prefrontal neurotransmitter release, and expression of genes related to serotonergic and dopaminergic signaling. A total of 31 dams and 196 offspring were used in the experiments and allocated to each experimental group. Results: Offspring in both HFD and 50% CDAHFD groups exhibited increased body weight and total cholesterol levels compared to controls, whereas plasma triglycerides were elevated a tendency in the 50% CDAHFD group. HFD offspring showed reduced spontaneous locomotor activity, and increased prefrontal serotonin release with altered serotonergic gene expression. Conversely, 50% CDAHFD offspring displayed increased exploratory behavior in the open field test alongside altered dopamine neuron-related genes, without locomotor or social deficits. Conclusions: Exposure of mothers to different high-fat diets has been suggested to cause specific changes in their offspring’s lipid profiles, which in turn modulate the serotonin and dopamine systems differently, leading to specific behavioral changes.

## 1. Introduction

Maternal nutritional status during the perinatal period plays a critical role in fetal development and has long-term effects on offspring health. Both maternal undernutrition and overnutrition have been shown to increase the risk of various diseases later in life, supporting the concept of the developmental origins of health and disease [1,2,3]. Among these nutritional disturbances, maternal obesity has become a major public health concern owing to its increasing prevalence worldwide [4]. Epidemiological studies suggest that maternal obesity may adversely affect the health of the offspring. Maternal obesity has been associated with an increased risk of metabolic abnormalities, including dyslipidemia, in the offspring [5]. Animal studies have also investigated the effects of exposure to maternal high-fat diet (HFD) during pregnancy and lactation on offspring development. Previous studies have reported that maternal HFD exposure induces obesity, dyslipidemia, and impaired glucose metabolism in offspring [6,7,8]. In addition, maternal metabolic disturbances during pregnancy have been associated with an increased risk of neurodevelopmental disorders in children, including autism spectrum disorder (ASD) and attention-deficit/hyperactivity disorder (ADHD) [9,10,11]. Animal studies have demonstrated impairments in social behavior, increased locomotor activity, and deficits in learning and memory that resemble certain features of neurodevelopmental disorders [12,13]. These findings suggest that maternal HFD exposure influences not only metabolic regulation but also brain function and behavior in offspring. Although the mechanisms underlying these behavioral alterations remain unclear, alterations in serotonergic and dopaminergic neurotransmission may represent one contributing factor. Sullivan et al. demonstrated that maternal high-fat diet exposure during pregnancy was associated with anxiety-like behavior in offspring and alterations in the serotonergic system in Japanese macaques [14]. Similarly, Vucetic et al. demonstrated that maternal HFD exposure during pregnancy altered dopaminergic signaling in offspring mice, including increased expression of dopamine transporters [15]. Collectively, these findings suggest that maternal HFD exposure influences offspring metabolism and behavior by altering monoaminergic neurotransmission. However, relatively few studies have examined the contribution of specific lipid fractions, rather than maternal obesity or HFD exposure as a whole, to offspring health outcomes. A prospective birth cohort study reported that maternal cholesterol levels during pregnancy were associated with the risk of ADHD in offspring [16]. Nevertheless, whether distinct lipid components, such as triglycerides and cholesterol, contribute differentially to metabolic and neurobehavioral outcomes in offspring remains unclear.

Triglycerides and cholesterol are major lipid components with distinct biological functions. Triglycerides primarily serve as an energy source and storage form of fatty acids, whereas cholesterol is an essential structural component of cell membranes and a precursor of steroid hormones and bile acids [17,18]. Altered triglyceride metabolism is a common feature of obesity and is closely associated with metabolic dysfunction [19]. However, despite their distinct physiological roles, whether triglycerides and cholesterol differentially influence metabolic and neurobehavioral development in offspring remains unclear. A choline-deficient, amino acid-defined high-fat diet (CDAHFD) is widely used to induce nonalcoholic steatohepatitis (NASH) [20]. We have previously reported the pathological characteristics of a CDAHFD-induced NASH mouse model exhibiting hepatic inflammation [21]. CDAHFD has been reported to induce metabolic alterations that are distinct from those observed with conventional HFDs, including elevated plasma triglyceride levels [22]. Therefore, comparing these dietary models may provide insight into the differential contributions of lipid metabolic alterations to offspring development. Given the distinct effects of conventional HFDs and CDAHFD on lipid metabolism, particularly triglyceride metabolism, we hypothesized that maternal lipid metabolic alterations would differentially influence metabolic and neurobehavioral outcomes in offspring.

Therefore, the present study compared the effects of these dietary models during pregnancy and lactation on lipid metabolism, behavior, and serotonergic and dopaminergic systems in offspring.

## 2. Materials and Methods

### 2.1. Experimental Animals

Pregnant ICR mice (embryonic day 11) were purchased from Japan SLC, Inc. (Shizuoka, Japan). Both the dams and their male offspring were used in this study. The breeding environment was maintained at a room temperature of 24 ± 1 °C and an ambient humidity of 55 ± 5%, with a 12-h light/dark cycle (light period: 07:00–19:00; dark period: 19:00–07:00). The present study was approved by the Animal Experiment Committee of Yokohama University of Pharmacy (approval number: 2023-019), and care was taken to ensure the welfare of the laboratory animals. Animal inclusion and exclusion criteria were established a priori. For behavioral testing (Open Field and 3-chamber social interaction tests) and in vivo microdialysis, animals exhibiting physical anomalies, severe illness, or mechanical failure of microdialysis probes/implants (e.g., cannula displacement, absence of stable baseline neurotransmitter recovery) were excluded from the analysis prior to unblinding. For molecular analyses (RT-qPCR), samples with poor RNA integrity (RIN < 7.0) or aberrant internal control amplification were excluded. No data points were excluded as statistical outliers unless verified as technical errors.

### 2.2. Experimental Diets

After 1 d of acclimatization, all dams were fed a standard rodent diet (Lab MR Stock, Nosan Corporation, Kanagawa, Japan) and water ad libitum. On embryonic day 12, the dams were randomly assigned to one of three dietary groups (Table 1): (1) the Control group, which received a standard diet (Lab MR Stock); (2) the HFD group, which received an HFD (High-Fat Diet 32; CLEA Japan, Tokyo, Japan); and (3) the 50% CDAHFD group, which received a 1:1 mixture of a CDAHFD (Research Diets INC, New Brunswick, NJ, USA) and the HFD. The experimental diets were provided ad libitum for 28 d, from embryonic day 12 to 3 weeks after birth (weaning). Total food intake was measured over the 28-d period. Thereafter, all dams were returned to the standard diet. A total of 31 dams and 196 offspring were used in the experiments and allocated to each experimental group. For all determinations, animals in each group originated from 1 to 10 different litters, and all male mice from each dam were used in a single experiment (the maximum number of offspring from a single litter included in any one experimental group was 12; Supplementary Table S1).

### 2.3. Plasma Lipid Measurements

When the offspring reached 3 weeks of age, blood samples were collected from the dams and their male offspring. The mice were anesthetized with isoflurane using a small animal anesthesia system (MK-AT210D; Muromachi Kikai, Tokyo, Japan), and blood was collected from the retro-orbital sinus using calibrated micropipettes (Drummond Scientific, Broomall, PA, USA). The blood samples were transferred to microtainer tubes (BD Microtainer; Becton Dickinson, Tokyo, Japan) and centrifuged at 15,000× *g* for 90 s at 4 °C. The plasma was then separated and stored. Plasma concentrations of triglycerides and total cholesterol were measured using commercial assay kits (LabAssay™ TG and LabAssay™ Cholesterol, respectively; FUJIFILM Wako Pure Chemical, Osaka, Japan).

### 2.4. Behavioral Assessment

Behavioral tests were performed in male offspring at 4–6 weeks of age. The tests were conducted in the following order: open field test (4–5 weeks age), spontaneous locomotor activity test (during the dark phase) (4–5 weeks age), and three-chamber social interaction test (6 weeks age). The behavioral tests were performed as previously described [23]. Each behavioral test was measured without blinding.

#### 2.4.1. Spontaneous Locomotor Activity

Spontaneous locomotor activity was measured using an activity-monitoring system (NS-AS01; Neuroscience, Tokyo, Japan). Mice were placed in individual cages, and locomotor activity was recorded at 1-h intervals using infrared sensors. Activity was monitored from 17:00 to 08:00 the following day. For the analysis, cumulative activity during the dark phase (19:00–07:00), which corresponds to the active period of the mice, was used.

#### 2.4.2. Open Field Test

The open field test was performed using an open-field apparatus (50 cm × 50 cm × 50 cm). Mice were placed in the center of the apparatus, and their behavior was recorded for 10 min. To eliminate odor cues, the apparatus was cleaned with 70% ethanol between trials. Behavioral analyses were performed using Viewer 2 software (version 2.2.0.17; Biobserve GmbH, Bonn, Germany). The open field was divided into a central zone (Zone 2; 25 cm × 25 cm) and a peripheral zone (Zone 1). The total distance traveled and the ratio of time spent in Zone 2 to that spent in Zone 1 were calculated.

#### 2.4.3. Three-Chamber Social Interaction Test

The three-chamber social interaction test was performed using a three-chamber apparatus (60 cm × 40 cm × 22 cm). The test mouse was placed in the center chamber and allowed to explore freely for 5 min (habituation phase). Subsequently, an unfamiliar mouse (stranger) was placed in a wire cage in one of the side chambers, while an empty wire cage was placed in the opposite side chamber. The test mouse was then returned to the center chamber and allowed to explore the apparatus for 10 min (test phase). The apparatus was cleaned with 70% ethanol between trials. The time spent in each chamber was measured to evaluate social preference. Behavioral analyses were performed using Viewer 2 software (version 2.2.0.17).

### 2.5. In Vivo Microdialysis

The day after completion of all behavioral tests, the mice were randomly assigned to one of two groups: one for microdialysis and the other for reverse transcription quantitative polymerase chain reaction (RT-qPCR). Microdialysis was performed as previously described [24,25]. The mice were anesthetized with isoflurane using a small animal anesthesia system (MK-AT210D) and fixed in a stereotaxic apparatus. A guide cannula was implanted targeting the prefrontal cortex (coordinates relative to bregma: anterior +1.9 mm, lateral +0.5 mm, ventral −0.8 mm). Following a 48-h recovery period, a dialysis probe was inserted and perfused with Ringer’s solution (147 mM NaCl, 2.4 mM CaCl2, 4.0 mM KCl, pH 7.0) at a flow rate of 1 μL/min. After 1 h of stabilization, dialysate samples were collected at 20-min intervals. Serotonin and dopamine concentrations in the dialysates were quantified using high-performance liquid chromatography with an electrochemical detector (HPLC-ECD; HTEC-500, Eicom, Kyoto, Japan).

### 2.6. Quantitative Real-Time RT-PCR

Following completion of the behavioral tests, the mice were euthanized by cervical dislocation, and the brain tissues were rapidly excised. The hippocampus, frontal cortex, and midbrain/pons were isolated. Total RNA was extracted using Isogen (Nippon Gene, Tokyo, Japan) according to the manufacturer’s protocol. The tissues were homogenized using a Polytron PT 1300 D (Central Scientific Commerce, Tokyo, Japan), and chloroform (Nacalai Tesque, Kyoto, Japan) was added for phase separation. After centrifugation, the aqueous phase was collected, and RNA was precipitated with isopropanol. cDNA was synthesized from the purified RNA using PrimeScript RT Master Mix (Perfect Real Time; Takara Bio, Shiga, Japan) at 37 °C for 15 min and 85 °C for 5 s. Quantitative real-time PCR was performed on a LightCycler 96 (Roche, Basel, Switzerland) using TB Green^®^ Premix Ex Taq™ II FAST qPCR (Takara Bio). The PCR conditions were as follows: initial denaturation at 95 °C for 30 s, followed by 40–50 cycles of 95 °C for 5 s and 60 °C for 10 s. Primer sequences are listed in Table 2.

*Gapdh*: *Mus musculus* glyceraldehyde-3-phosphate dehydrogenase; *Tph*: *Mus musculus* tryptophan hydroxylase; *Htr1a*: *Mus musculus* 5-hydroxytryptamine (serotonin) 1A receptor; *Htr2a*: *Mus musculus* 5-hydroxytryptamine (serotonin) 2A receptor; *Th*: *Mus musculus* tyrosine hydroxylase; *Drd3*: *Mus musculus* dopamine D3 receptor; *Drd4*: *Mus musculus* dopamine D4 receptor; *Drd5*: *Mus musculus* dopamine D5 receptor; *Bdnf*: *Mus musculus* Brain-derived neurotrophic factor.

### 2.7. Statistical Analysis

Statistical analyses were performed using linear mixed-effects models or one-way analysis of variance (ANOVA), as appropriate. Plasma lipid concentrations in the offspring, open field test data, and three-chamber social interaction test data were analyzed using linear mixed-effects models, with dietary group as a fixed effect and dam ID as a random intercept to account for the non-independence of offspring from the same litter. Pairwise comparisons among dietary groups were performed using estimated marginal means, with *p*-values adjusted using Tukey’s method. Dam intake data, post hoc pairwise comparisons were performed using Tukey’s method.

Hourly changes in spontaneous locomotor activity were analyzed using a linear mixed-effects model, with dietary group, time, and their interaction as fixed effects and dam ID and individual ID as random intercepts. Pairwise comparisons among dietary groups at each time point and for total locomotor activity over the 12-h observation period were performed using estimated marginal means, with *p*-values adjusted using Tukey’s method.

Microdialysis data and gene expression data obtained by real time RT-PCR were analyzed using one-way ANOVA because the limited number of dams made the application of mixed-effects models difficult. For microdialysis data, post hoc pairwise comparisons were performed using Tukey’s method. For gene expression data, pairwise comparisons among groups were performed using estimated marginal means, and *p*-values were adjusted using the Benjamini–Hochberg procedure separately for dopamine-related genes, serotonin-related genes, and BDNF expression in the three brain regions. Statistical significance was set at an adjusted *p* < 0.05.

All statistical analyses were performed with EZR (Saitama Medical Center, Jichi Medical University, Saitama, Japan), which is a graphical user interface for R (The R Foundation for Statistical Computing, Vienna, Austria). Differences were considered statistically significant at *p* < 0.05 and *p* < 0.01.

## 3. Results

### 3.1. Plasma Lipid Levels

**Figure 1:**
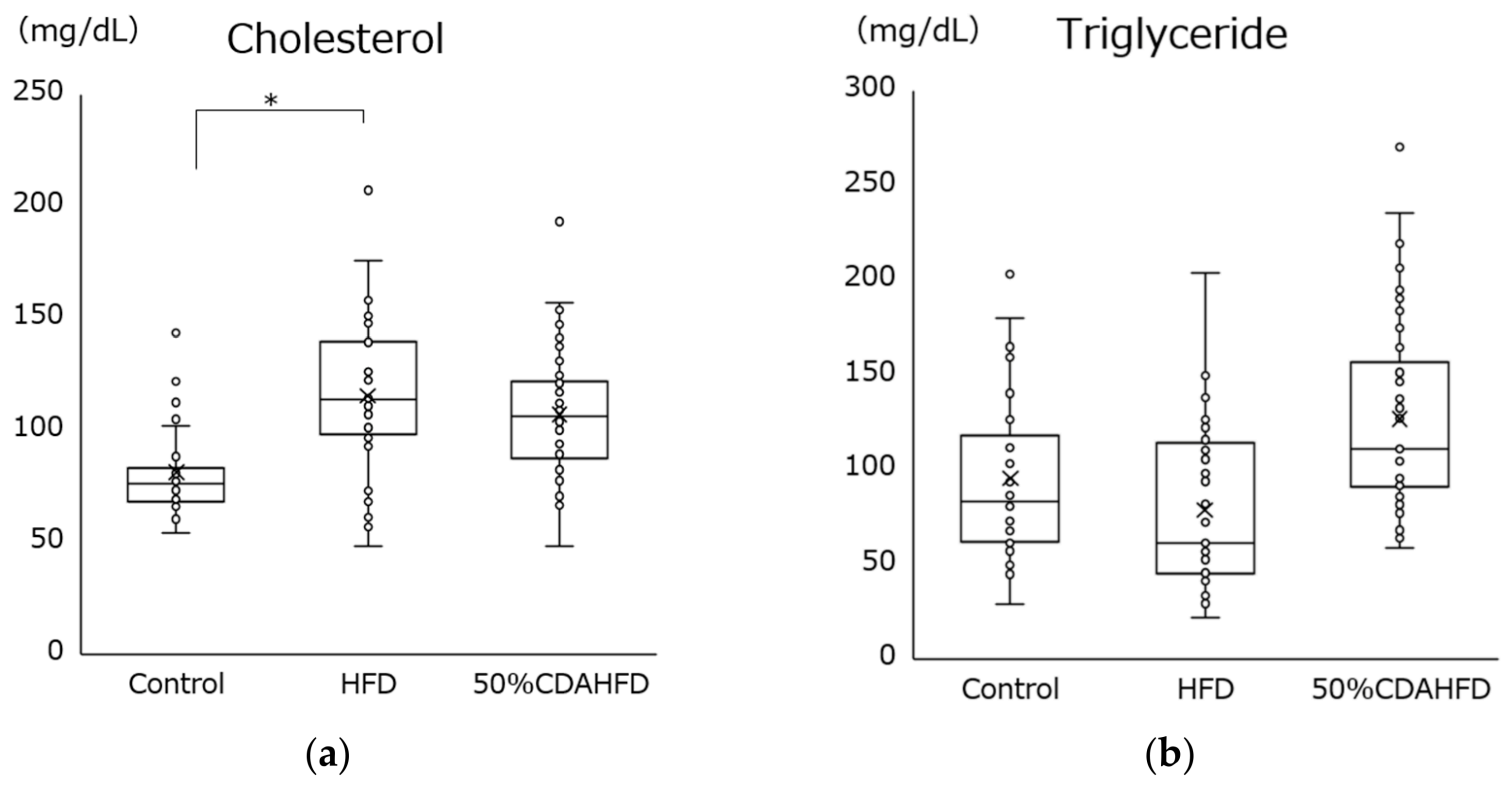

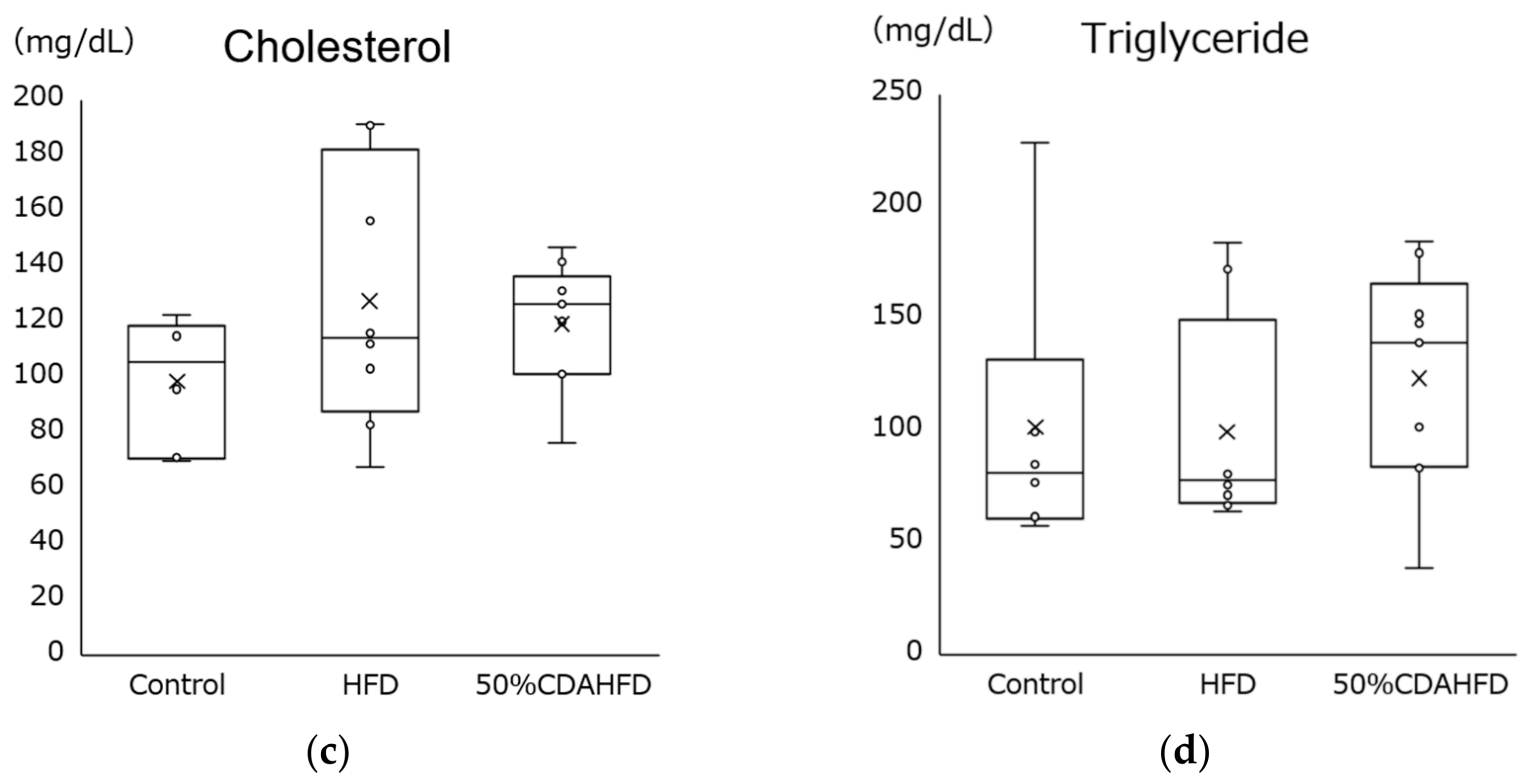
Effects of maternal diet on plasma lipid metabolic markers in offspring. Plasma levels of total cholesterol and triglycerides in offspring (**a**,**b**) and dams (**c**,**d**). Plasma cholesterol levels were significantly higher in the HFD group than in the Control group. In contrast, plasma triglyceride levels were tendency higher in the 50% CDAHFD group than in the HFD group. Offspring Control (*n* = 30), HFD (*n* = 37), 50%CDAHFD (*n* = 46). Dams Control (*n* = 6), HFD (*n* = 8), 50%CDAHFD (*n* = 9). * *p* < 0.05, Tukey–Kramer test. Box-and-whisker plots show the median and 25th and 75th percentiles; whiskers indicate the minimum and maximum values, and crosses indicate the mean.

The effects of maternal diet on plasma lipid levels in dams and their offspring are shown in Figure 1a,b. In the offspring, plasma total cholesterol levels were 81.1 ± 20.4 mg/dL in the Control group, 115.3 ± 32.4 mg/dL in the HFD group, and 107.0 ± 28.2 mg/dL in the 50% CDAHFD group, with a significant increase observed in the HFD group compared with the Control group (*p* < 0.05, Figure 1a). Meanwhile, plasma triglyceride levels were 95.1 ± 47.2 mg/dL in the Control group, 78.5 ± 42.8 mg/dL in the HFD group, and 126.4 ± 50.5 mg/dL in the 50% CDAHFD group, showing a trend toward higher levels in the 50% CDAHFD group compared with the HFD group (*p* = 0.0836, Figure 1b). Similar trends in plasma lipid levels were observed in the dams, although no significant differences were detected among the groups (Figure 1c,d).

### 3.2. Food and Caloric Intake of Dams

**Figure 2:**
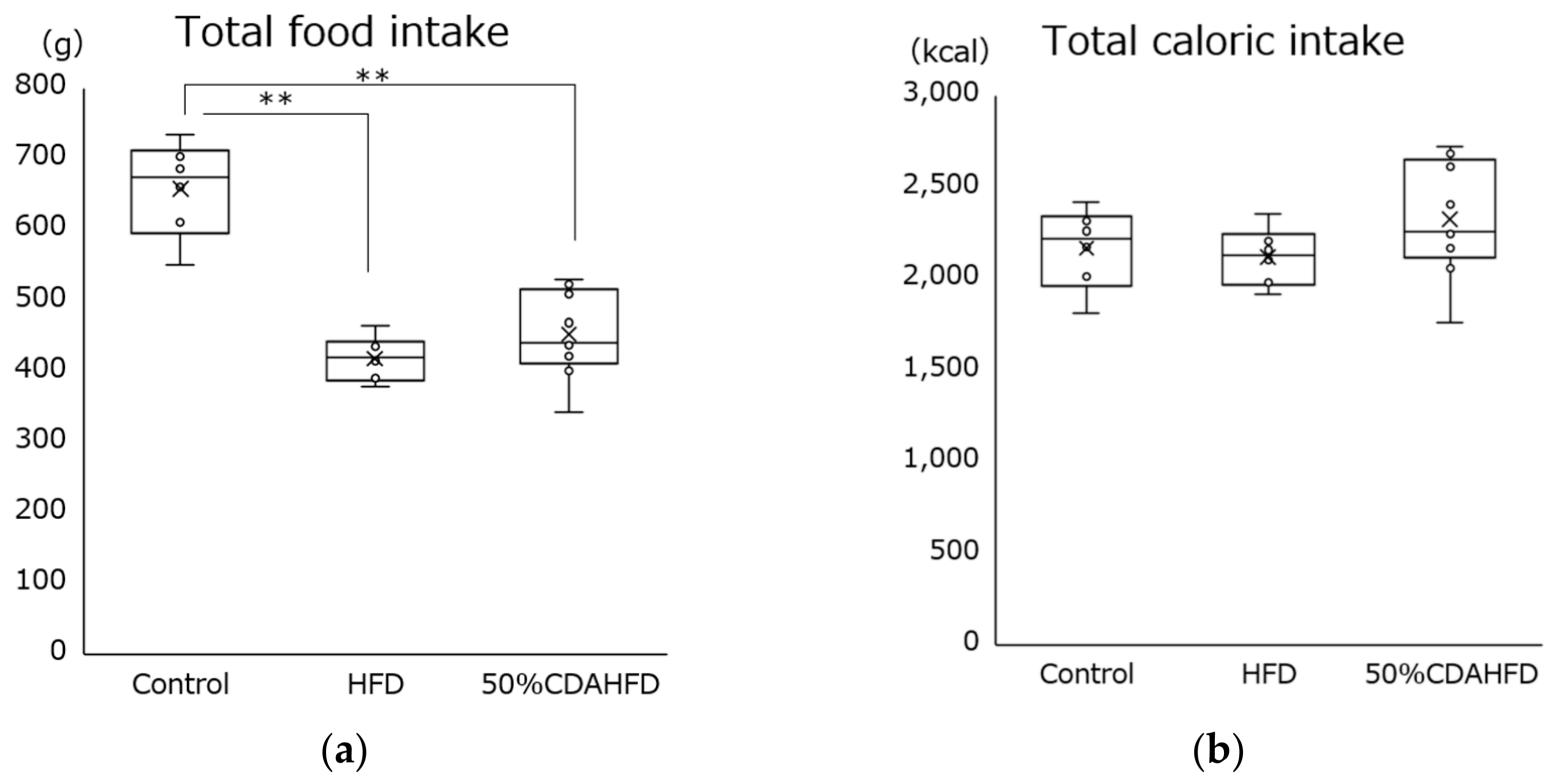
Effects of maternal diet on food and caloric intake of dams. (**a**) Total food intake and (**b**) total caloric intake of dams during the 28-d experimental period. Total food intake during the 28-d period was significantly lower in both the HFD and 50% CDAHFD groups than in the Control group, whereas no significant differences were observed in total caloric intake among the groups. Control (*n* = 6), HFD (*n* = 6), 50%CDAHFD (*n* = 9). ** *p* < 0.01, Tukey–Kramer test. Box-and-whisker plots show the median and 25th and 75th percentiles; whiskers indicate the minimum and maximum values, and crosses indicate the mean.

The total food and caloric intake of the dams during the 28-d experimental period are shown in Figure 2a,b. Total food intake was 657.3 ± 67.3 g in the Control group, 417.3 ± 31.0 g in the HFD group, and 451.9 ± 61.7 g in the 50% CDAHFD group. The HFD and 50% CDAHFD groups exhibited significantly lower food intake than the Control group (*p* < 0.01; Figure 2a). In contrast, no significant differences in total caloric intake were observed among the three groups (Control: 2163.2 ± 221.6 kcal; HFD: 2118.0 ± 157.5 kcal; 50% CDAHFD: 2350.0 ± 320.8 kcal).

### 3.3. Body Weight of Male Offspring at 4 Weeks of Age

**Figure 3:**
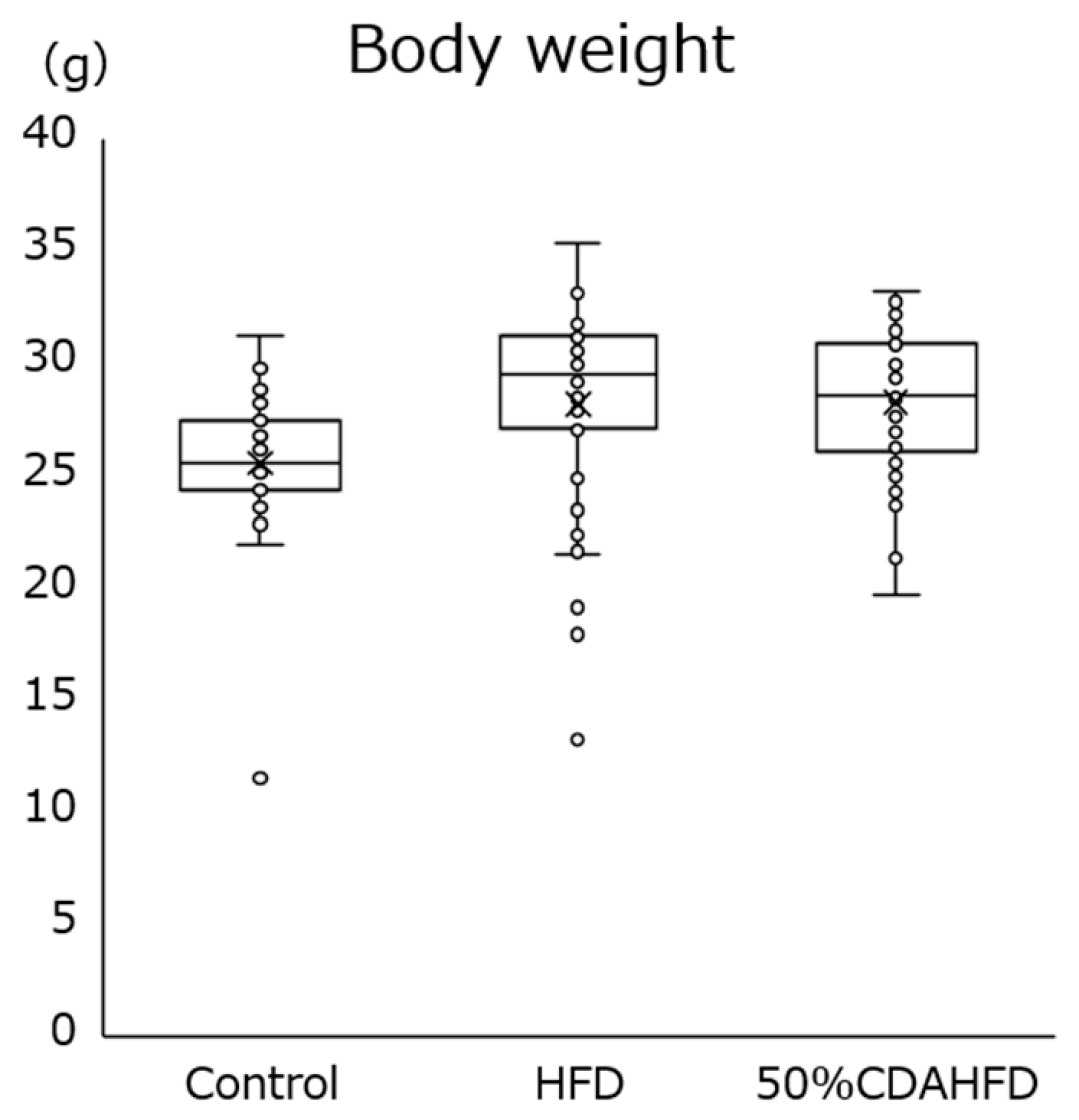
Effects of maternal diet on body weight of offspring. Body weight did not differ significantly among the groups. Control (*n* = 36), HFD (*n* = 48), 50%CDAHFD (*n* = 75). Tukey–Kramer test. Box-and-whisker plots show the median and 25th and 75th percentiles; whiskers indicate the minimum and maximum values, and crosses indicate the mean.

The effects of maternal diet on the body weight of male offspring at 4 weeks of age are shown in Figure 3. Body weight did not differ significantly among the groups (25.5 ± 3.2 g in the Control group, 28.1 ± 4.4 g in the HFD group, and 28.2 ± 3.0 g in the 50% CDAHFD group).

### 3.4. Open Field Test

**Figure 4:**
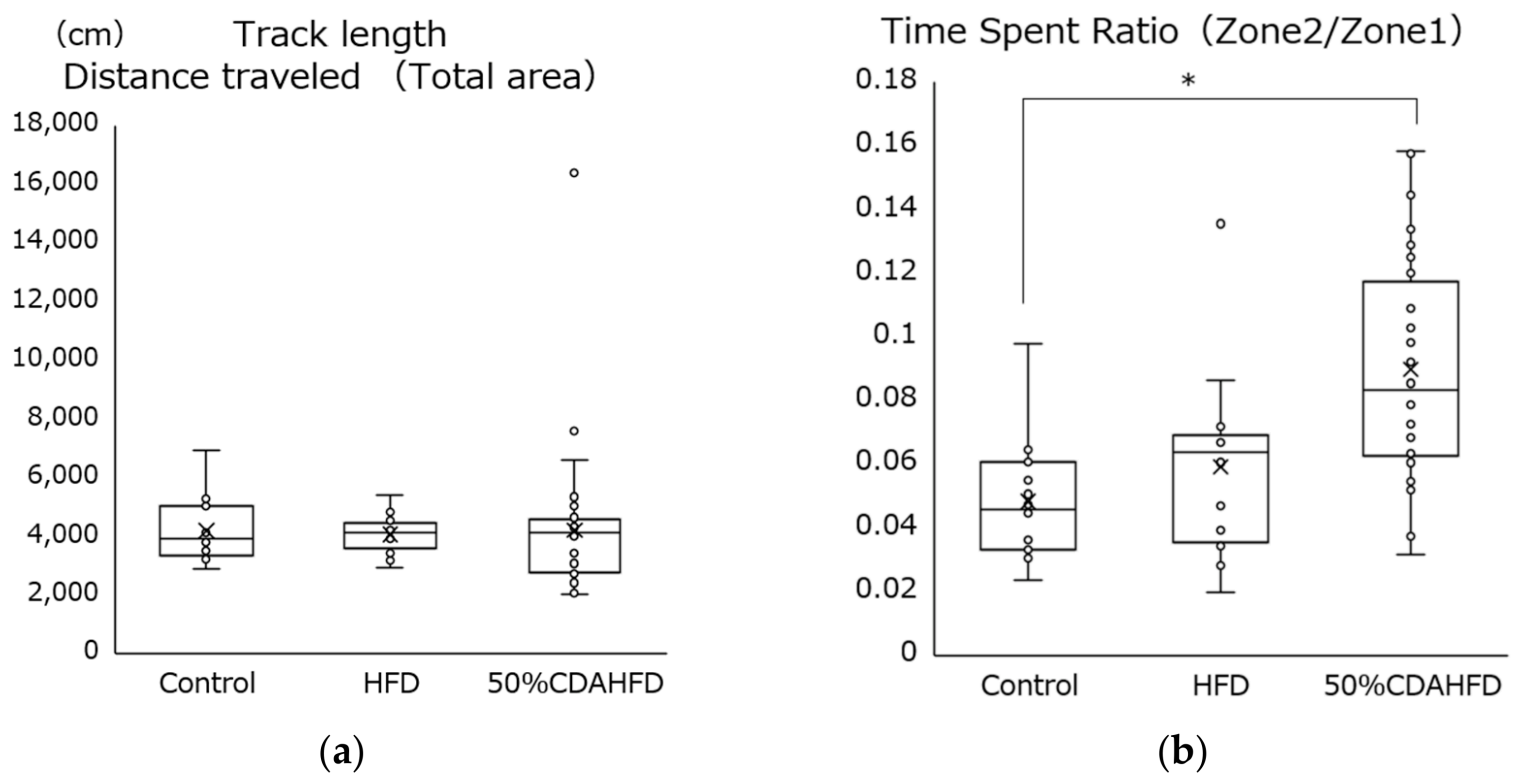
Behavioral analysis in the open field. (**a**) Total distance traveled. (**b**) Ratio of time spent in the central zone (Zone 2) to that spent in the peripheral zone (Zone 1). Open field test results. No significant differences were observed in total distance traveled, whereas the percentage of time spent in the central zone was significantly higher in the 50% CDAHFD group than the Control group. Control (*n* = 15), HFD (*n* = 14), 50%CDAHFD (*n* = 36). * *p* < 0.05, Tukey–Kramer test. Box-and-whisker plots show the median and 25th and 75th percentiles; whiskers indicate the minimum and maximum values, and crosses indicate the mean.

The effects of maternal diet on the behavior of male offspring in the open field test are shown in Figure 4a,b. No significant differences in total distance traveled were observed among the three groups. However, the 50% CDAHFD group showed a significantly higher ratio of time spent in the central zone (Zone 2) to that spent in the peripheral zone (Zone 1) than the Control group (*p* < 0.05; Figure 4b).

### 3.5. Spontaneous Locomotor Activity During the Dark Phase

**Figure 5:**
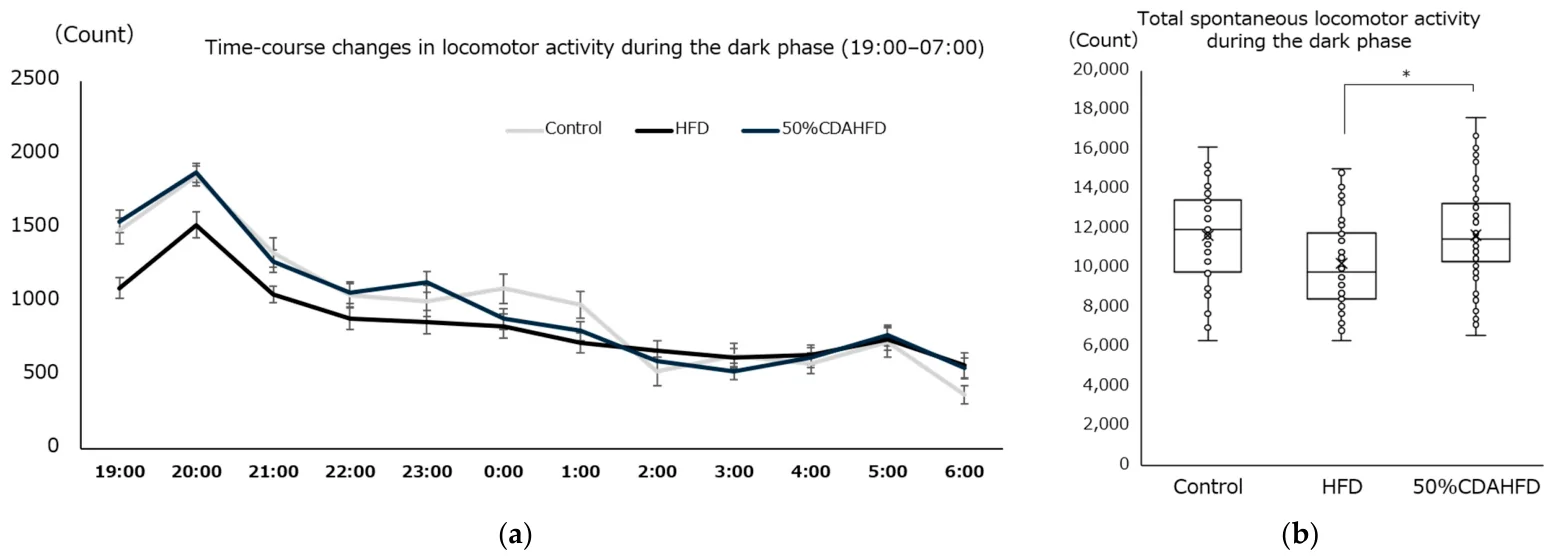
Effects of maternal diet on spontaneous locomotor activity in male offspring. (**a**) Time-course changes in locomotor activity during the dark phase (19:00–07:00). (**b**) Total spontaneous locomotor activity during the dark phase. Measurement of spontaneous locomotor activity during the dark phase revealed significantly decreased activity in the HFD group than in the 50%CDAHFD group, whereas the 50% CDAHFD group showed values comparable to those in the Control group. Control (*n* = 33), HFD (*n* = 47), 50%CDAHFD (*n* = 67). * *p* < 0.05, Tukey–Kramer test. Line graph shows mean  ±  SE. Box-and-whisker plots show the median and 25th and 75th percentiles; whiskers indicate the minimum and maximum values, and crosses indicate the mean.

The effects of maternal diet on spontaneous locomotor activity in male offspring during the dark phase are shown in Figure 5a,b. Total spontaneous locomotor activity during the dark phase in the HFD group was significantly lower than that in the 50% CDAHFD group and exhibited a trend toward lower levels compared with the Control group (*p* < 0.05 vs. Control; *p* = 0.0586 vs. 50% CDAHFD; Figure 5b).

### 3.6. Three-Chamber Social Interaction Test

**Figure 6:**
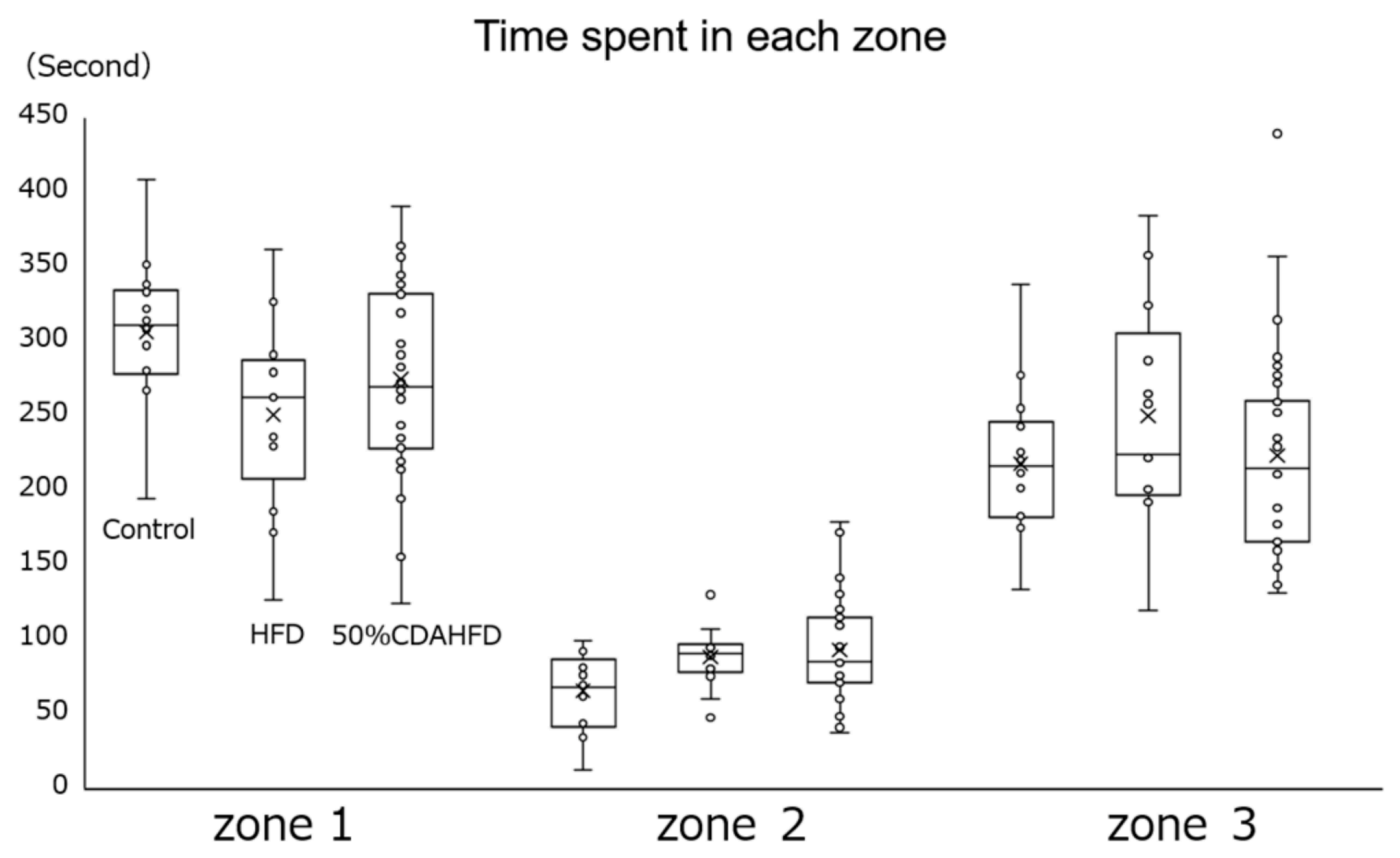
Behavioral analysis in the three-chamber social interaction tests. Data represent the time spent in each zone by male offspring with Zone 1 containing a stranger mouse. No significant differences in time spent in each zone were observed among the groups. Control (*n* = 15), HFD (*n* = 14), 50%CDAHFD (*n* = 36). Tukey–Kramer test. Box-and-whisker plots show the median and 25th and 75th percentiles; whiskers indicate the minimum and maximum values, and crosses indicate the mean.

The effects of maternal diet on social behavior in male offspring assessed using the three-chamber social interaction test are shown in Figure 6. No significant differences in time spent in each zone were observed among the groups.

### 3.7. Neurotransmitter Release in the Prefrontal Cortex

**Figure 7:**
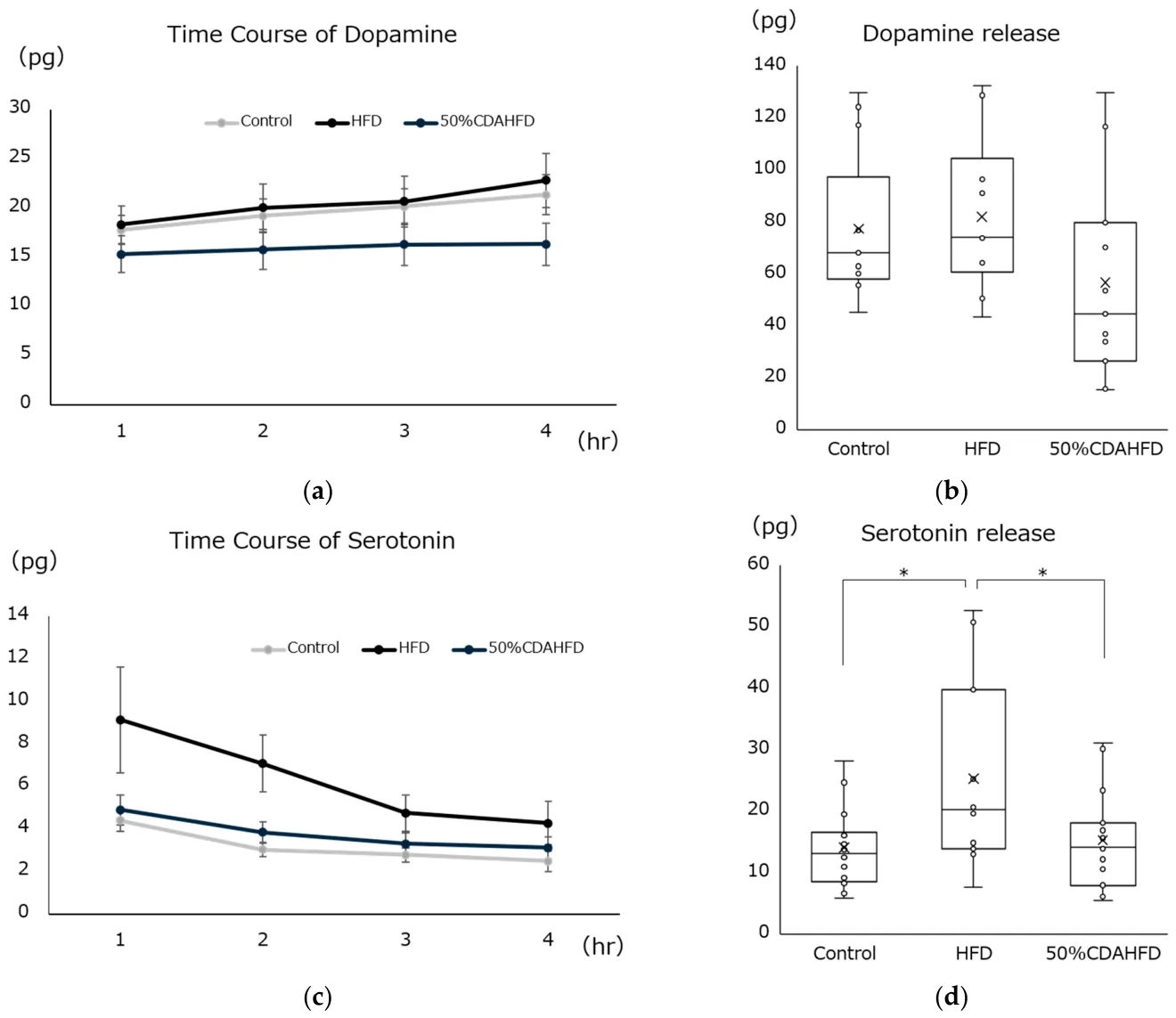
Neurotransmitter release in the prefrontal cortex of male offspring. Dopamine (**a**,**b**) and serotonin (**c**,**d**) released from the prefrontal cortex after microdialysis. Data were collected and measured every 20 min for 4 h. Regarding neurotransmitter levels in the prefrontal cortex, total dopamine release showed a decreasing trend in the 50% CDAHFD group compared with the other groups. In contrast, total serotonin release showed a significant increase in the HFD group compared to the other groups, whereas the 50% CDAHFD group showed values comparable to those of the control group. Dopamine: Control (*n* = 19), HFD (*n* = 10), 50%CDAHFD (*n* = 17), Serotonin: Control (*n* = 19), HFD (*n* = 11), 50%CDAHFD (*n* = 15). * *p* < 0.05, Tukey–Kramer test. Box-and-whisker plots show the median and 25th and 75th percentiles; whiskers indicate the minimum and maximum values, and crosses indicate the mean.

The effects of maternal diet on dopamine and serotonin release in the prefrontal cortex of male offspring were evaluated using in vivo microdialysis (Figure 7a–d). Total dopamine release over 4 h was comparable between the HFD and Control groups, whereas the 50% CDAHFD group tended to have lower levels than both the Control and HFD groups (Figure 7b). In contrast, total serotonin release was significantly higher in the HFD group than in the Control group, whereas this increase was significantly attenuated in the 50% CDAHFD group (*p* < 0.05; Figure 7d).

### 3.8. Real Time RT-PCR

**Figure 8:**
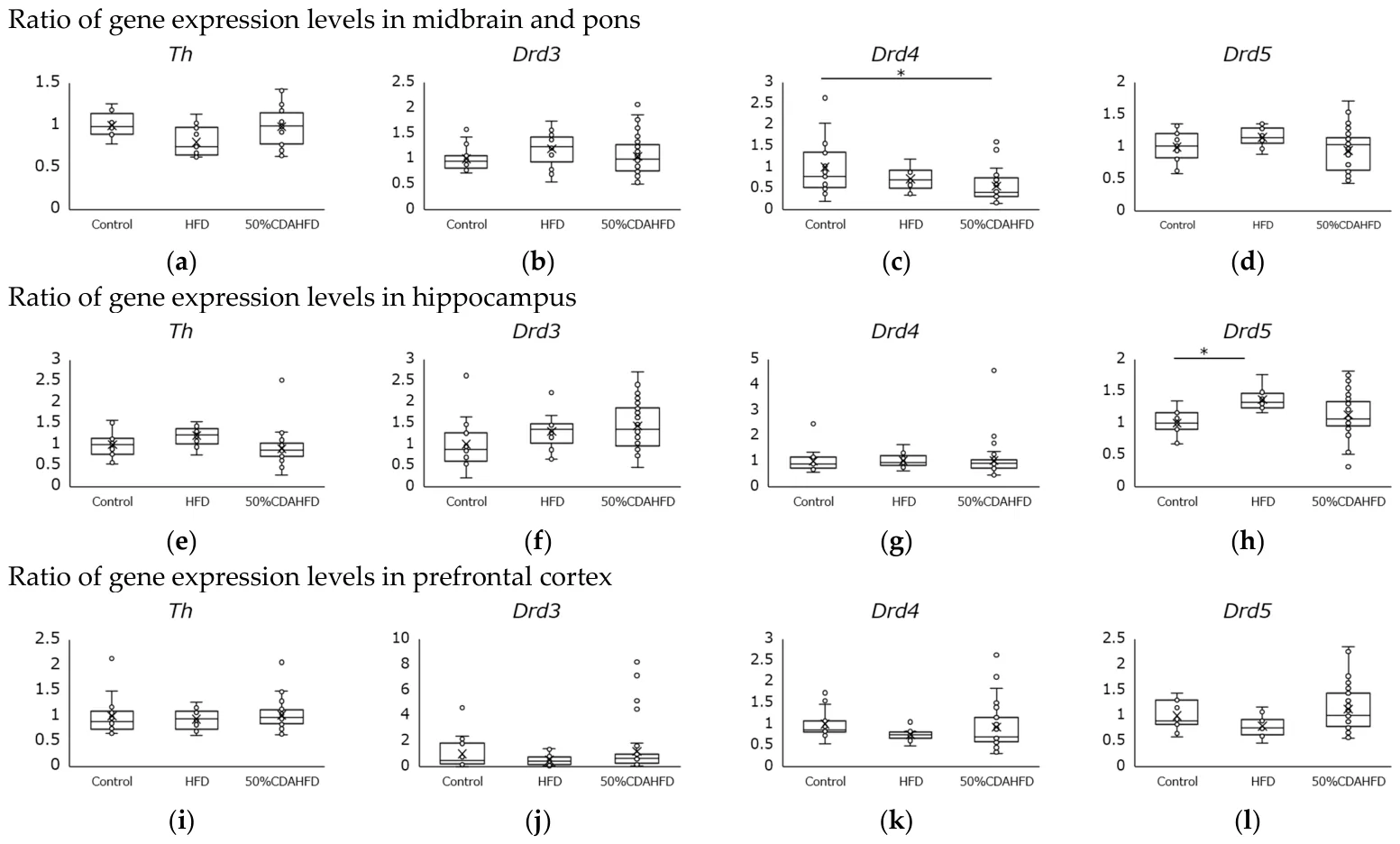
Expression levels of genes related to the dopaminergic system. Expression levels of genes in the midbrain/pons (**a**–**d**), hippocampus (**e**–**h**), and prefrontal cortex (**i**–**l**). *Th*, tyrosine hydroxylase; *Drd3*, dopamine D3 receptor; *Drd4*, dopamine D4 receptor; *Drd5*, dopamine D5 receptor. In the midbrain/pons, *Drd4* expression was significantly decreased in the 50% CDAHFD group compared with the Control group. In the hippocampus, *Drd5* expression was significantly increased in the HFD group compared with the Control group. In the prefrontal cortex, No significant differences were observed in any of the genes. Midbrain/pons: Control (*n* = 8), HFD (*n* = 13), 50%CDAHFD (*n* = 16), hippocampus and prefrontal cortex: Control (*n* = 15), HFD (*n* = 13), 50%CDAHFD (*n* = 37). * *p* < 0.05, Tukey–Kramer test. Box-and-whisker plots show the median and 25th and 75th percentiles; whiskers indicate the minimum and maximum values, and crosses indicate the mean.

**Figure 9:**
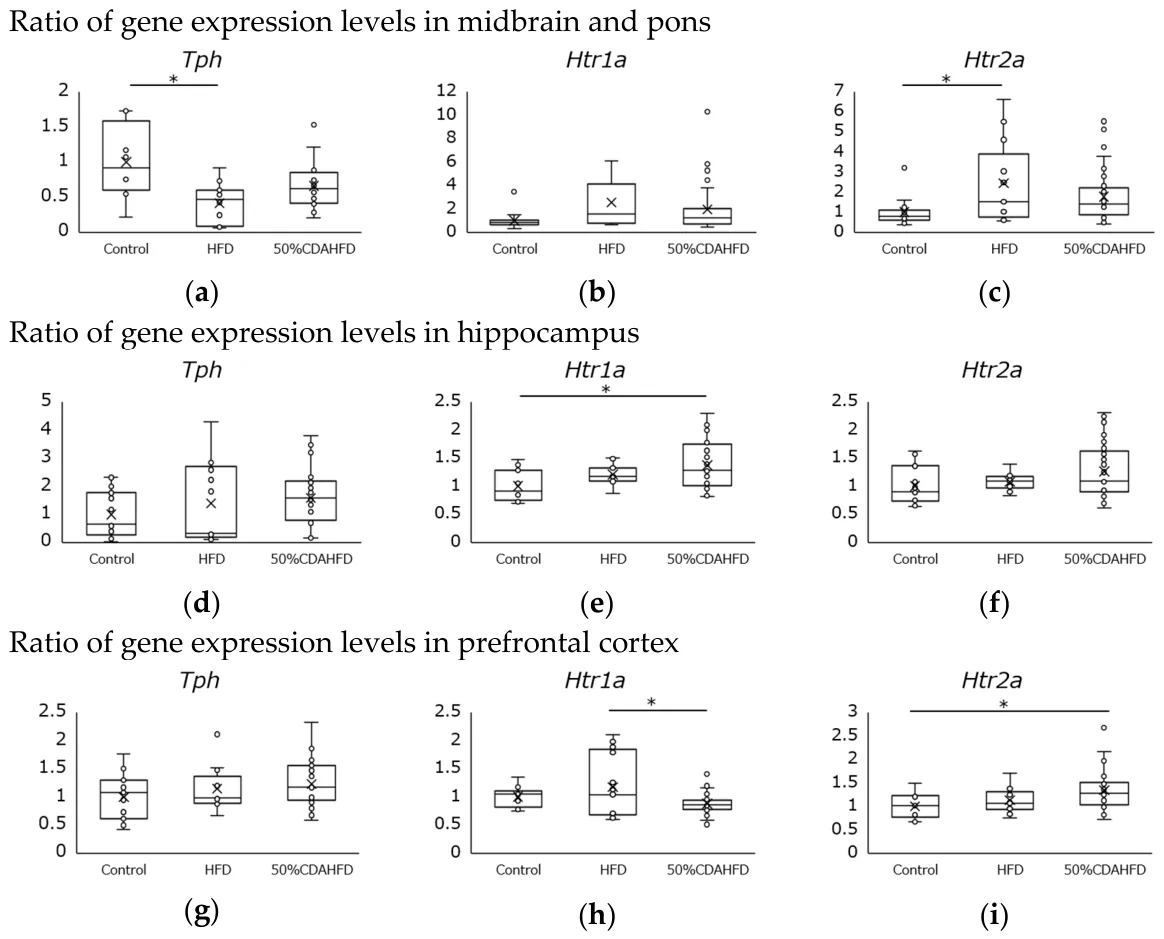
Expression levels of genes related to the serotonergic system. Expression levels of genes in the midbrain/pons (**a**–**c**), hippocampus (**d**–**f**), and prefrontal cortex (**g**–**i**). *Tph*, tryptophan hydroxylase; *Htr1a*, serotonin 1A receptor; *Htr2a*, serotonin 2A receptor. In the midbrain/pons, the gene expression levels of *Tph*, the rate-limiting enzyme in serotonin synthesis, were significantly decreased in the HFD group compared with the Control group. In addition, the expression level of the *Htr2a* was significantly increased in the HFD group compared with the Control group. In the hippocampus, the expression level of *Htr1a* was significantly increased in the 50% CDAHFD group compared with the Control group. In the prefrontal cortex, the expression level of *Htr1a* showed an increasing trend in the HFD group compared with the Control group and was significantly increased compared with the 50% CDAHFD group. Furthermore, the expression level of *Htr2a* was significantly increased in the 50% CDAHFD group compared with the Control group. Midbrain/pons: Control (*n* = 8), HFD (*n* = 13), 50%CDAHFD (*n* = 16), hippocampus and prefrontal cortex: Control (*n* = 15), HFD (*n* = 13), 50%CDAHFD (*n* = 37). * *p* < 0.05, Tukey–Kramer test. Box-and-whisker plots show the median and 25th and 75th percentiles; whiskers indicate the minimum and maximum values, and crosses indicate the mean.

The mRNA expression levels of the genes in the midbrain/pons, hippocampus, and prefrontal cortex were measured by RT-PCR. Figure 8 and Figure 9 illustrates the effects of maternal feeding with a normal diet, HFD, or 50% CDAHFD on the expression of genes related to the dopaminergic and serotonergic systems in the respective brain regions of male offspring. Gene expression levels were normalized to *Gapdh* and expressed as relative ratios, with the value for the Control group set to 1.

Figure 8 shows the gene expression levels of dopamine-related markers. In the midbrain/pons, *Drd4* expression was significantly decreased in the 50% CDAHFD group compared with the Control group (*p* < 0.05; Figure 8c). In the hippocampus, *Drd5* expression was significantly increased in the HFD group compared with the Control group (*p* < 0.05; Figure 8h). In the prefrontal cortex, No significant differences were observed in any of the genes. No significant differences were observed for *Drd1* and *Drd2* (Supplementary Figure S1).

Figure 9 illustrates the gene expression levels of serotonin-related markers. In the midbrain/pons, the gene expression levels of *Tph*, the rate-limiting enzyme in serotonin synthesis, were significantly decreased in the HFD group compared with the Control group (*p* < 0.05; Figure 9a). In addition, the expression level of the *Htr2a* was significantly increased in the HFD group compared with the Control group (*p* < 0.05; Figure 9c). In the hippocampus, the expression level of *Htr1a* was significantly increased in the 50% CDAHFD group compared with the Control group (*p* < 0.05; Figure 9e). In the prefrontal cortex, the expression level of *Htr1a* showed an increasing trend in the HFD group compared with the Control group and was significantly increased compared with the 50% CDAHFD group (*p* < 0.05; Figure 9h). Furthermore, the expression level of *Htr2a* was significantly increased in the 50% CDAHFD group compared with the Control group (*p* < 0.05; Figure 9i).

**Figure 10:**
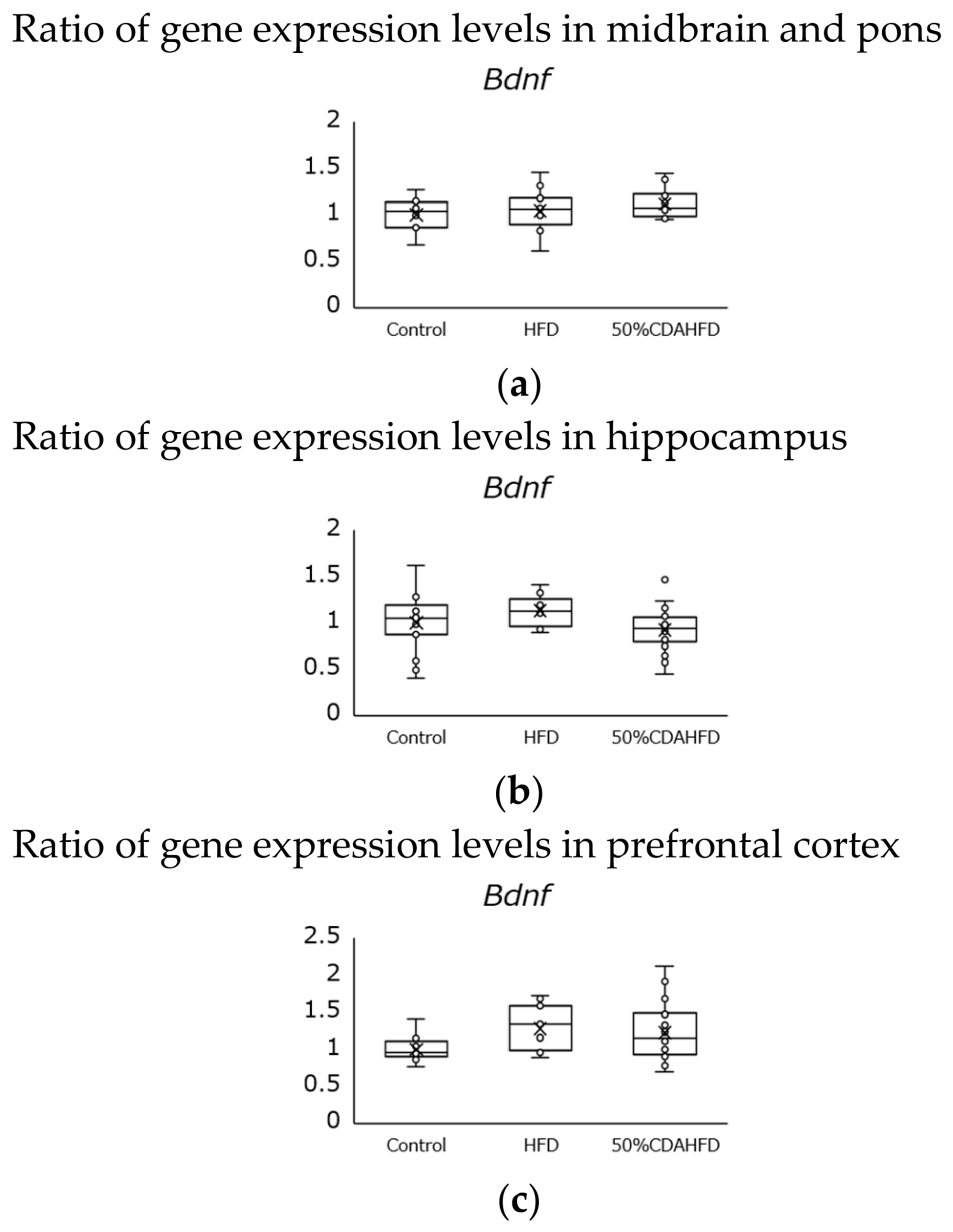
Expression levels of genes related to the Brain-derived neurotrophic factor. Expression levels of genes in the midbrain/pons (**a**), hippocampus (**b**), and prefrontal cortex (**c**). *Bdnf*, Brain-derived neurotrophic factor. In the midbrain/pons, no significant differences in *Bdnf* gene expression levels were observed among the groups (**a**). In the prefrontal cortex, a tendency increase was observed in the HFD group compared with the Control group (*p* = 0.059; (**b**)). In the hippocampus, a tendency decrease was observed in the 50% CDAHFD group compared with the HFD group (*p* = 0.059; (**c**)). Midbrain/pons: Control (*n* = 8), HFD (*n* = 13), 50%CDAHFD (*n* = 16), hippocampus and prefrontal cortex: Control (*n* = 15), HFD (*n* = 13), 50%CDAHFD (*n* = 37). Tukey–Kramer test. Box-and-whisker plots show the median and 25th and 75th percentiles; whiskers indicate the minimum and maximum values, and crosses indicate the mean.

Figure 10 illustrates the gene expression levels of Brain-derived neurotrophic factor (*Bdnf*). In the midbrain/pons, no significant differences in *Bdnf* gene expression levels were observed among the groups (Figure 10a). In the prefrontal cortex, a tendency increase was observed in the HFD group compared with the Control group (*p* = 0.059; Figure 10b). In the hippocampus, a tendency decrease was observed in the 50% CDAHFD group compared with the HFD group (*p* = 0.059; Figure 10c).

## 4. Discussion

Maternal nutritional status plays a critical role in offspring development. In particular, excessive lipid exposure during pregnancy has been reported to increase the risk of neurodevelopmental disorders in offspring. However, evidence regarding the effects of specific lipid fractions on offspring neurodevelopment remains limited. In the present study, we demonstrate for the first time that maternal exposure to either an HFD or a CDAHFD, both of which induce distinct lipid metabolic profiles, affects lipid metabolism in offspring and induces behavioral alterations by modulating the serotonergic and dopaminergic system.

To evaluate the differential effects of the two maternal HFDs on lipid metabolism in offspring, plasma cholesterol and triglyceride levels were measured. A previous study reported that maternal HFD consumption beginning 1 week before pregnancy increased plasma cholesterol levels in offspring [26]. Consistent with these findings, offspring born to dams fed a conventional HFD exhibited significantly higher plasma cholesterol levels than those in the Control group. Interestingly, offspring born to dams fed the 50% CDAHFD exhibited tendency higher plasma triglyceride levels, suggesting that this dietary model induces a lipid metabolic profile distinct from that induced by the conventional HFD. The lipid content of the feeds used in this study differed significantly between the control diet and the HFD and 50% CDAHFD diets, although the lipid content of the HFD and 50% CDAHFD diets were comparable. Unlike the conventional HFD, CDAHFD induces a distinct pattern of lipid metabolic alterations through choline and methionine deficiency. Choline and methionine deficiency has been reported to impair very-low-density lipoprotein synthesis and secretion, resulting in hepatic triglyceride accumulation [22,27]. Therefore, the elevated plasma triglyceride levels observed in the 50% CDAHFD group may reflect lipid metabolic alterations that differ from those induced by the conventional HFD. Furthermore, no significant difference in body weight was observed in the offspring of either the HFD group or the 50% CDAHFD group, nor was there any difference in maternal calorie intake between the two groups.

Embryonic day 12 is a critical stage of brain development in mice during which environmental factors can have long-lasting effects on neurodevelopment. Accordingly, and based on previous studies, including the valproic acid-induced ASD model [28], maternal dietary intervention in the present study was initiated at embryonic day 12. To determine whether the distinct plasma lipid profiles among the three groups were associated with behavioral alterations, behavioral analyses were performed in offspring at 4–6 weeks of age. The groups did not differ significantly in total distance traveled during the 10-min open field test, offspring from the 50% CDAHFD group spent a significantly greater proportion of time in the center area than those from the Control group, whereas no significant difference was observed between the HFD and Control groups. Increased exploration of the center area in the open field test has been reported in animal models exhibiting reduced anxiety-like behavior and in several schizophrenia-related mouse models [29]. Similarly, increased center-area activity has been reported in dopamine receptor knockout mice, suggesting that dopaminergic neurotransmission may contribute to this behavioral phenotype [30]. Therefore, the altered exploratory behavior observed in the 50% CDAHFD group may be associated with changes in dopaminergic function. Interestingly, this behavioral phenotype was observed only in offspring with elevated plasma triglyceride levels, suggesting that triglyceride-related metabolic alterations during early development may influence neurodevelopmental processes and subsequent behavioral outcomes.

Assessment of spontaneous locomotor activity further revealed distinct behavioral effects of the two maternal dietary models. Compared with offspring in the Control group, offspring in the HFD group exhibited tendency reduced spontaneous locomotor activity, whereas no such impairments were observed in the 50% CDAHFD group. Uçeyler et al. reported that serotonin transporter-deficient mice exhibit reduced locomotor activity, suggesting that impaired serotonergic neurotransmission may contribute to decreased spontaneous locomotor behavior [31]. In our previous study, we demonstrated that chronic administration of dexamethasone, a synthetic glucocorticoid derived from cholesterol, reduced spontaneous voluntary locomotor activity during the active phase in mice and that this behavioral impairment was ameliorated by treatment with the selective serotonin reuptake inhibitor fluvoxamine [32]. We also reported that ovariectomy-induced reductions in spontaneous locomotor activity are associated with alterations in serotonergic neurotransmission and that modulation of the serotonergic system ameliorates this behavioral impairment [23,33,34]. Taken together, these findings suggest that the reduced spontaneous locomotor activity observed in the HFD group may be mediated, at least in part, by alterations in cholesterol metabolism and subsequent changes in serotonergic neurotransmission. Maternal cholesterol is transported to the fetus through the placenta, whereas maternal triglycerides contribute to the fetal lipid supply following placental hydrolysis to free fatty acids [35]. Although cholesterol and triglycerides do not readily cross the blood–brain barrier (BBB), cholesterol-derived oxysterols and triglyceride-derived free fatty acids have been reported to penetrate the BBB and influence brain lipid metabolism [36,37]. Interestingly, offspring in the 50% CDAHFD group did not exhibit the reductions in spontaneous locomotor activity observed in the HFD group. This finding raises the possibility that triglyceride-related metabolic alterations may differentially influence neurobehavioral development, thereby modifying the behavioral consequences of maternal HFD exposure. However, unlike conventional high-fat diets, the 50% CDAHFD used in this study is deficient in choline and methionine, so it is not fully clear whether the observed neurobehavioral developmental changes are particularly related to changes in the lipid profile, and further investigation is needed.

To further elucidate these behavioral changes, we measured extracellular dopamine and serotonin levels in the brain using microdialysis and quantified the expression of related genes using real-time RT-PCR. Quantitative analysis of dopamine-related genes revealed that the expression level of *Drd4* in the midbrain/pons was significantly lower in the 50% CDAHFD group than in the Control group, whereas that of *Drd5* in the prefrontal cortex was significantly higher in the 50% CDAHFD group than in the HFD group. It has been reported that mutations or defects in the dopaminergic system can cause behavioral changes, such as an increase or decrease in exploratory behavior [38]. In this context, Berland et al. reported that triglycerides, when administered intracerebrally, inhibit the dopaminergic system [39]. In the present study, no changes in exploratory behavior were observed in the HFD group, in which plasma triglyceride levels remained unchanged. In contrast, the 50% CDAHFD group, which exhibited elevated plasma triglyceride levels, showed significant changes in exploratory behavior. These findings suggest that plasma lipids may be involved in these behavioral changes. Therefore, the increased central exploratory behavior observed in the open field test in the 50% CDAHFD group may be associated with a trend toward elevated plasma triglyceride levels. Alterations in the dopaminergic system may be one potential mechanism underlying this behavioral change, although the relationship between triglyceride levels and dopaminergic function remains to be elucidated.

In contrast, extracellular serotonin levels in the prefrontal cortex were elevated in the HFD group, whereas those in the 50% CDAHFD group were comparable to those in the Control group. Regarding serotonin synthesis, the gene expression level of *Tph*, the rate-limiting enzyme in the midbrain/pons, a region containing the raphe nuclei, was significantly lower in the HFD group. Furthermore, the expression of the *Htr1a* in the prefrontal cortex, a projection area of these neurons, showed an increasing trend. These results suggest that the sustained elevation of extracellular serotonin levels in the prefrontal cortex may have induced the upregulation of Serotonin 1AR, a Gi-protein-coupled receptor, which may, in turn, have led to a decrease in tryptophan hydroxylase expression in the midbrain/pons through a negative feedback mechanism. In the prefrontal cortex, the expression level of *Htr2a* was significantly higher in the HFD group than in the Control group. Halberstadt et al. reported that administration of a Serotonin 2AR agonist reduces activity levels in mice [40]. The decrease in spontaneous motor activity observed in the HFD group in this study may be related to the serotonergic system in the prefrontal cortex.

Furthermore, a novel finding of this study is that maternal HFD and 50% CDAHFD intake differentially alters plasma lipid concentrations in the offspring. Notably, the triglyceride-to-cholesterol ratio differed significantly between the two groups. Specifically, the triglyceride-to-cholesterol ratio in the 50% CDAHFD group was comparable to that in the control group (Control group, 1.20; 50% CDAHFD group, 1.23), whereas this ratio was markedly lower in the HFD group (HFD group, 0.77). Here, Chang et al. reported that prenatal exposure to an HFD enhanced neurogenesis and affected behavioral outcomes in the offspring [41]. In addition, maternal HFD exposure has been reported to alter BDNF expression in offspring [42,43]; however, the direction of these changes, whether an increase or a decrease, has not been consistent across experimental conditions. In the present study, HFD exposure tendency increased *Bdnf* expression in the prefrontal cortex, whereas hippocampal *Bdnf* expression was tendency lower in the 50% CDAHFD group than in the HFD group. These differences in *Bdnf* expression may be partly related to differences in plasma lipid balance. Moreover, differences in plasma lipid balance may have contributed to differences in serotonin release, potentially through alterations in *Bdnf* expression.

Although the biological significance of this lipid balance remains unclear, these findings raise the possibility that the relative balance between triglycerides and cholesterol, rather than the absolute concentration of either lipid alone, may influence neurobehavioral development.

Several limitations of the present study should be noted. First, our analyses focused exclusively on the adult stage of the offspring, without evaluating neurochemical or behavioral parameters during the embryonic or early postnatal periods. Second, because only male offspring were used in this study, potential sex differences could not be evaluated. Third, in the microdialysis and RT-PCR analyses, litter effects could not be accounted for due to the limited number of dams. Fourth, because the dams were fed the experimental diets from pregnancy through lactation, it remains unclear whether the observed changes are a direct primary consequence of fetal programming or secondary adaptations that developed throughout postnatal life.

## 5. Conclusions

In summary, maternal consumption of two different HFDs during pregnancy alters offspring lipid profiles and influences neurological and behavioral development, suggesting that triglyceride concentrations are associated with neurobehavioral changes.

## Figures and Tables

**Table 1:** Composition and energy content of experimental diets.

Group	Composition Details	Choline (mg/kg)	Methionine (%)	Energy (kcal/100 g)	Fat (% kcal)
Control (*n* = 9) ^1^	Lab MR Stock	1820.0	0.26	329.1	3.9
HFD (*n* = 9)	High-Fat Diet 32	1479.6	0.26	507.6	56.7
50%CDAHFD (*n* = 13)	CDAHFD: HFD = 1: 1 (*w*/*w*)	739.8	0.4~0.55	513.8	59.35
CDAHFD ^2^		0	0.105	520	62

^1^ The *n* number in parentheses indicates the number of dam mice. ^2^ CDAHFD: A choline-deficient, amino acid-defined high-fat diet.

**Table 2:** Primers for each marker used in real-time RT-PCR.

Gene	Forward Primer	Reverse Primer
*Gapdh*	agcttgtcatcaacgggaag	tttgatgttagtggggtctcg
*Tph*	cacagttcagatcccctctaca	gaacgtggcctaggagttca
*Htr1a*	caggcaggcatggatatgtt	ctccagggacgttgtggt
*Htr2a*	ctgctgggtttccttgtcat	gtaaatccagacggcacagag
*Th*	cccaagggcttcagaagag	atgaaccttggggacgtg
*Drd3*	ttcaataagggccaggtttctgtc	tccatgtgtctggccaggag
*Drd4*	ctatgtcaacagtgccctcaacc	agcgcactctgcacacagtc
*Drd5*	tggtttccataagcgcacagta	gagcactggcacttgtggtttc
*Bdnf*	agtctccaggacagcaaagc	tgcaaccgaagtatgaaataacc

## Data Availability

The data that support the findings will be available in The University of Osaka Institutional Knowledge Archive (OUKA) at https://ir.library.osaka-u.ac.jp/repo/ouka/all/?lang=1 following an embargo from the date of publication to allow for commercialization of research findings.

## Supplementary Materials

The following supporting information can be downloaded at: https://www.mdpi.com/article/10.3390/nu18213589/s1, Table S1: Summary of Litters and Number of Offspring Used per Experiment. Figure S1: Expression levels of genes related to the dopaminergic system.

## Abbreviations

The following abbreviations are used in this manuscript:

HFD	High-Fat Diet
CDAHFD	Choline-Deficient, Amino acid-defined High-Fat Diet
ASD	Autism Spectrum Disorder
ADHD	Attention-Deficit/Hyperactivity Disorder
NASH	nonalcoholic steatohepatitis
RT-qPCR	reverse transcription quantitative polymerase chain reaction
GAPDH	glyceraldehyde-3-phosphate dehydrogenase
TPH	tryptophan hydroxylase
Serotonin 1AR	5-hydroxytryptamine 1A receptor
Serotonin 2AR	5-hydroxytryptamine 2A receptor
TH	tyrosine hydroxylase
dopamine 1R	dopamine 1 receptor
dopamine 2R	dopamine 2 receptor
dopamine 3R	dopamine 3 receptor
dopamine 4R	dopamine 4 receptor
dopamine 5R	dopamine 5 receptor
BDNF	Brain-derived neurotrophic factor
