# Supplementary material for: Maternal HFD and CDAHFD Exposure from Pregnancy to Lactation Differentially Affect Lipid Profiles and Behavior in Offspring Mice
Source: Nutrients. 2026 Oct 29;18(21):3589. doi: 10.3390/nu18213589 (PMC13765132; doi:10.3390/nu18213589)
Supplement: Supplementary file 1 [file nutrients-18-03589-s001.zip › nutrients-4611281-supplementary.pdf]

Supplementary Materials

Supplementary Table S1

The total numbers of dams were 9 in the Control group, 9 in the HFD group, and 13 in the 50% CDAHFD group. As not all dams and their offspring were used for every experiment, the sample sizes varied across analyses depending on the experimental procedures performed.

Experiments were conducted in batches. Specific experimental procedures were conducted in separate batches to ensure consistent ages of the offspring at the time of testing and due to practical constraints, including limited personnel and resources.

The numbers of dams and offspring included in each experiment are summarized below.

Table S1. Summary of Litters and Number of Offspring Used per Experiment.

**(Dams)**	**Body weight**		**Plasma Lipid Levels**		**Spontaneous Locomotor Activity**		**Open Field Test and Social Interaction Test**	
Group	Dams (n)	Offspring (n)	Dams (n)	Offspring (n)	Dams (n)	Offspring (n)	Dams (n)	Offspring (n)
Control	6	36	6	30	6	36	6	33
HFD	7	48	8	37	7	48	7	47
50％CDAHFD	10	75	9	46	10	75	10	67

	**Microdialysis Dopamine**		**Microdialysis Serotonin**		**qPCR midbrain and pons**		**qPCR hippocampus and prefrontal cortex**	
Group	Dams (n)	Offspring (n)	Dams (n)	Offspring (n)	Dams (n)	Offspring (n)	Dams (n)	Offspring (n)
Control	4	19	4	19	1	8	2	15
HFD	3	10	3	11	2	13	2	13
50％CDAHFD	4	17	4	15	2	16	4	37

Figure S1 shows the gene expression levels of dopamine-related markers. No significant differences were observed in any of the genes.

Ratio of gene expression levels in midbrain and pons

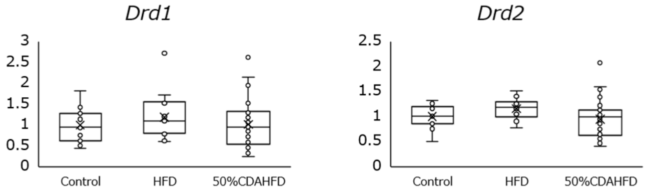

(**a**) (**b**)

Ratio of gene expression levels in hippocampus

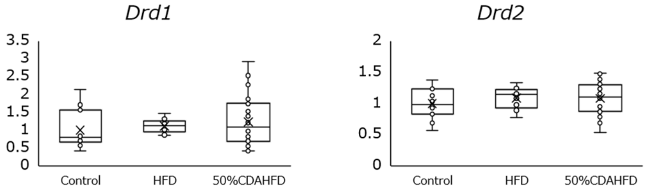

(**c**) (**d**)

Ratio of gene expression levels in prefrontal cortex

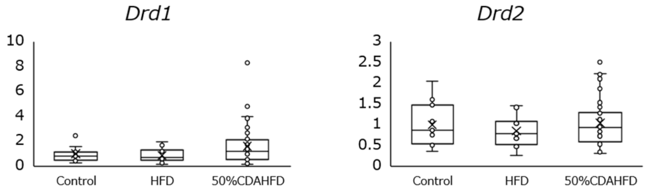

(e) (**f**)

Figure S1. Expression levels of genes related to the dopaminergic system. Expression levels of genes in the midbrain/pons (**a**,b), hippocampus (**c,d**), and prefrontal cortex (**e,f**). *Drd1*, dopamine D1 receptor; *Drd2*, dopamine D2 receptor. In the midbrain/pons, No significant differences were observed in any of the genes. Midbrain/pons: Control ($n = 8$), HFD ($n = 13$), 50%CDAHFD ($n = 16$), hippocampus and prefrontal cortex: Control ($n = 15$), HFD ($n = 13$), 50%CDAHFD ($n = 37$). * $p < 0.05$, Tukey–Kramer test. Box-and-whisker plots show the median and 25th and 75th percentiles; whiskers indicate the minimum and maximum values, and crosses indicate the mean.
